# Association between the Mediterranean Diet and Vitamin C and the Risk of Head and Neck Cancer

**DOI:** 10.3390/nu15132846

**Published:** 2023-06-23

**Authors:** Constanza Saka-Herrán, Tanya Pereira-Riveros, Enric Jané-Salas, José López-López

**Affiliations:** 1 Department of Odontostomatology, Faculty of Medicine and Health Sciences (Dentistry), University of Barcelona, 08970 Barcelona, Spain; constanzasakah@gmail.com (C.S.-H.); tanyapereirariveros@gmail.com (T.P.-R.); enricjanesalas@ub.edu (E.J.-S.); 2 Oral Health and Masticatory System Group (Bellvitge Biomedical Research Institute) IDIBELL, Faculty of Medicine and Health Sciences (Dentistry), University of Barcelona, 08970 Barcelona, Spain; 3 Head Service of the Medical-Surgical Area of the Dentistry Hospital, Faculty of Medicine and Health Sciences (Dentistry), Bellvitge Campus, University of Barcelona, 08970 Barcelona, Spain

**Keywords:** head and neck cancer, head and neck neoplasms, cancer, risk, Mediterranean diet, diet, Mediterranean, ascorbic acid, vitamin C, diet, antioxidants

## Abstract

Diet may modulate the risk of head and neck cancer (HNC) through antioxidant and anti-inflammatory effects. To date, there is limited evidence regarding the effects of the Mediterranean diet on HNC risk. The purpose of the study was to assess the association between Mediterranean diet adherence, type of diet, and vitamin C and the risk of HNC. A case–control study was conducted at the Dentistry Hospital, University of Barcelona, including 101 cases of HNC and 101 controls matched by age and sex. Dietary habits were assessed using a 14-question Mediterranean diet score that classified the type of diet into healthy diet (10–14 points), regular diet (5–9 points), and unhealthy diet (≤4 points). Multivariate logistic regression models were used to assess the association between Mediterranean diet adherence, type of diet, and vitamin C and the risk of HNC. Higher adherence to the Mediterranean diet was significantly associated with a lower risk of HNC (OR = 0.88, 95% CI: 0.79–0.98). A healthy diet (OR = 0.29, 95% CI: 0.10–0.84) and vitamin C intake (OR = 0.25, 95% CI: 0.10–0.62) were strongly associated with lower odds of HNC. Moderate egg intake was the only type of food significantly associated with a lower risk of HNC. Dietary patterns that emphasize a high intake of antioxidant and anti-inflammatory bioactive components may have a protective effect on the risk of HNC.

## 1. Introduction

Head and neck cancer (HNC) includes a group of neoplasms that are associated with high morbidity and mortality. In 2020, cancers of the oral cavity, pharynx, and larynx accounted for 4.6% of all new cases and 4.5% of all new deaths worldwide [1]. HNC is highly prevalent in South Central Asia and Melanesia, with the highest incidence rate worldwide in both sexes, reflecting the popularity of betel nut chewing [1]. Over the last 50 years, the epidemiology of head and neck cancer has changed significantly, as smoking-related HNC has decreased in incidence while human-papillomavirus-related cancers have increased [2]. While tobacco smoking, alcohol drinking, and human papilloma virus infection are well established risk factors for HNC, the impact of other lifestyle factors, such as physical activity and dietary patterns, are not yet fully elucidated [3]. There is limited, suggestive evidence that consuming non-starchy vegetables and choosing healthy dietary patterns might decrease the risk of HNC, and inconclusive evidence exists for grains, fruits, legumes, dietary fiber, red meat, fish, eggs, dairy products, total fat, and vitamins [3]. 

Overall, dietary patterns that emphasize foods from plant sources, such as vegetables, fruits, whole grains, and legumes are associated with a lower cancer risk [4]. The protective effect is attributed to different mechanisms that complement and overlap with antioxidant, anti-inflammatory, anti-angiogenic, and anti-proliferative effects [5]. Through their anti-inflammatory and antioxidant effects, they neutralize free radicals and reactive oxygen species, counteracting the inflammatory process and subsequent oxidative damage to DNA, which plays a significant role in the onset and progression of cancer [5,6]. The bioactive components that mainly act as antioxidants are vitamin C, vitamin E, β-carotene, and the minerals selenium and manganese, which are mainly found in fruits, vegetables, and whole grains [7].

The effects of several bioactive components on HNC risk have been previously assessed. Vitamin C has been associated with a lower risk of cancers of the oral cavity/pharynx (OR = 0.54, 95% CI: 0.45–0.65) and larynx (OR = 0.52, 95% CI: 0.40–0.68) [8]. Vitamin E has also been associated with a lower risk of HNC. Specifically, ≥13.5 mg/day of vitamin E was associated with lower odds of oral/pharyngeal cancer (OR = 0.59, 95% CI: 0.49–0.71) and laryngeal cancer (OR = 0.67, 95% CI: 0.54–0.83) [9]. On the other hand, results from a systematic review found no association between the general intake of carotenoids and HNC risk (OR = 0.48, 95% CI: 0.19–1.27) [10]. However, when analyzed by specific carotenoid groups, the authors observed a significant beneficial effect of β-carotene on oral cancer (OR = 0.54, 95% CI: 0.37–0.80) and laryngeal cancer risk (OR = 0.43, 95% CI: 0.24–0.77) [10].

It has been suggested that a dietary pattern with a higher antioxidant vitamin and fiber intake is associated with a decreased risk of oral/pharyngeal (OR = 0.57, 95% CI: 0.41–0.78) and laryngeal cancer (OR = 0.62, 95% CI: 0.37–1.00) [11]. In contrast, a dietary pattern based on animal products and refined cereals and a diet rich in saturated fats has been associated with an increased risk of laryngeal cancer (OR = 1.51, 95% CI: 1.1–2.1 and OR = 1.8, 95% CI: 1.40–2.30, respectively) [11]. Similarly, a dietary pattern based on high fruit and vegetable intake and low red meat consumption has been associated with a lower HNC risk (OR = 0.34, 95% CI: 0.24–0.49) [12]. Specifically, the inverse association was strongest for green vegetables, allium vegetables, citrus fruits, pears, and apples. Conversely, red and processed meats were associated with an increased risk of HNC (OR = 1.40, 95% CI: 1.13–1.74) [12].

The Mediterranean diet is recognized as part of the cultural heritage of France, Italy, Greece, Spain, and Morocco and is characterized by a high intake of fruits, vegetables, legumes, whole grains, nuts, and olive oil; a moderate intake of fish, dairy products, and ethanol (red wine); and a low intake of red meat [13]. The positive relationship between the Mediterranean diet and cancer is attributed to the high contents of antioxidants and anti-inflammatory components which prevent cellular DNA from oxidative stress [13]. Vegetables, olive oil, and red wine have antioxidant and anti-inflammatory properties due to their high concentration of polyphenols. Fruits and vegetables likewise have a high concentration of carotenoids, vitamins, folates, and flavonoids recognized for their antioxidant properties. A low intake of red meat contributes to moderating the harmful effects of high-temperature meat cooking and also reduces the intake of animal fats [13]. 

Nevertheless, there is limited evidence regarding the effects of Mediterranean diet adherence on head and neck cancer risk. Filomeno et al. [14] reported that an adherence between 66.7 and 100% was associated with an 80% risk reduction of oral and pharyngeal cancer compared with an adherence of 0–57.8% (OR = 0.20, 95% CI: 0.14–0.28). The results of a systematic review also showed that a higher adherence to the Mediterranean diet was associated with a lower risk of HNC (RR = 0.56, 95% CI: 0.44–0.72) [15]. The authors, however, suggest interpreting the results with caution due to the low certainty of evidence [15].

The results provided in this report are part of a study which aimed to assess the effects of non-classical risk factors and potential protective factors on head and neck cancer risk. Regarding the effects of diet on HNC, the hypothesis of the research was that a higher adherence to the Mediterranean diet, a healthy diet, and vitamin C intake will be associated with a lower risk of HNC. The aim was to assess the association between Mediterranean diet adherence, type of diet, and vitamin C intake and the risk of head and neck cancer.

## 2. Materials and Methods

### 2.1. Design and Study Population

The detailed methodology of this research has been published previously [16]. A hospital-based case–control study was designed. Patients ≥ 18 years who underwent treatment at the Dental Hospital, University of Barcelona (HOUB), Bellvitge campus between 2018 and 2022 made up the study population. The Clinical Research Ethics Committee of the HOUB approved the research project in March 2018 (approval code: 05-2018). 

### 2.2. Definition of Cases and Controls

Cases were patients treated at the HOUB in the Master of Dentistry in Oncology and Immunocompromised Patients program who had been diagnosed with HNC by histological confirmation (ICD C00-C14, C30-C32) between 2018 and 2022. In the study, incident cases of HNC diagnosed since 2014 were considered. Older than 18 years old, able to respond to a questionnaire, and able to give their informed consent were the inclusion criteria. Those who could not be examined or surveyed due to medical needs were excluded, as were those who had previously been diagnosed with any other type of cancer. 

Controls were patients who underwent treatment at the HOUB as part of the Master of Medicine, Surgery, and Oral Implantology program or who visited the Dentistry degree program between 2014 and 2022. They were chosen consecutively based on eligibility criteria and matched to cases in a 1:1 ratio by age (10 years) and sex. Patients ≥ 18 years without a history of cancer who could complete a questionnaire and provide their informed consent were eligible for inclusion. Patients with medical restrictions that prevented them from being examined or surveyed, those who had a history of HNC or another type of cancer, and those who had potentially malignant oral lesions were excluded from the study. 

### 2.3. Data Source, Identification of Cases and Controls, and Recruitment

The electronic medical records of the GESDEN^®^ program, which have been used in the HOUB since 2014 (index date), were used to identify the cases and controls. Patients who met the selection criteria were subsequently contacted and invited to participate in the study. For those patients who provided their informed consent, a one-day visit was planned at the HOUB.

### 2.4. Sample Size

Based on the results of the systematic review by Morze et al. [15], a total sample size of 156 participants (78 cases and 78 controls) was calculated to estimate an OR of 0.56 (95% CI: 0.44–0.72) for the association between adherence to the Mediterranean diet and the risk of HNC, with a 5% significance level, 80% power, and a 2-tailed test. However, since the research project also included other main objectives, the largest sample size, calculated at 196 participants (98 cases and 98 controls) was used [16]. The G*Power 3.1 program was used to calculate the sample size.

### 2.5. Data Collection

A structured survey designed for research purposes was used to collect the data. Sociodemographic information, tobacco and alcohol histories, dietary preferences, physical activity levels, and comorbidities were all recorded. Additionally, HNC patients were requested to give their medical records to the HOUB detailing their diagnosis and treatment on the appointed day. In accordance with the 10th revision of the International Classification of Diseases (ICD C00-C14, C30-C32), the topographic location of the cancer as well as the date of diagnosis, TNM stage, HPV status, treatment received, and disease recurrence were recorded.

### 2.6. Exposure

Mediterranean Diet: Dietary habits were evaluated using a questionnaire on the Mediterranean diet that was adapted from the Spanish Society of Atherosclerosis. The questionnaire consisted of 14 questions with a yes/no response and categorized the type of diet as unhealthy (≤4 points), regular (5–9 points), or healthy (10–14 points) [17]. The questions were 1. Do you eat vegetables at least 6 days per week? 2. Do you eat 2 or more servings of fruit per day? 3. Do you eat whole-grain foods (bread, cereal, rice, etc.) at least once a day? 4. Do you eat legumes 2 or more days per week? 5. Do you eat nuts 2 or more days per week? 6. Do you consume olive oil or seed oil (corn, sunflower, soybean, etc.) daily? 7. Do you eat fresh or frozen fish (tuna, sardine, salmon, mackerel, anchovy, etc.) and/or seafood 2 or more times per week? 8. Do you eat less than 4 eggs per week? 9. Do you eat red meat or sausages less than 3 times per week? 10. Do you drink whole milk and dairy products more than 2 days per week? 11. Do you add salt to meals at the table? 12. Do you eat industrial pastries more than 2 days per week? 13. Do you drink industrial soft drinks (not light) more than 2 days per week? 14. Do you have a moderate alcohol intake? (Moderate alcohol consumption in men was equal to 2 standard beverage units (1 SBU = 10 mL ethanol) per day (i.e., 2 glasses of wine, two-fifths of beer, or 1 glass of spirits), while it was equal to 1 SBU per day (i.e., 1 glass of wine, one-fifth of beer, or half a glass of spirits) for women and those over 65 years.) Positive answers (Yes) were given a +1 value and negative responses (No) a value of −1, except for questions 10, 11, 12, and 13, where the positive answer was given a −1 value. 

Vitamin C: Vitamin C intake was assessed using three questions designed for the study purposes. The questions were 1. Do you eat at least 1 serving of fruit such as strawberry, kiwi, orange, grapefruit, or red/green bell pepper per day? 2. Do you consume at least 1 glass of natural fruit juice per day? 3. Do you regularly take vitamin C supplements? Each question had a yes/no answer. Participants who answered yes to any of the 3 questions were considered vitamin C users (Question 1 = Yes OR Question 2 = Yes OR Question 3 = Yes).

### 2.7. Covariates

Cases and controls were matched for sex and age in 3 categories: 45–54 years, 55–64 years, and ≥65 years. Without education, primary education, secondary education, and higher education were used to categorize levels of education. According to the level of monthly income, monthly salaries were divided into 3 categories: low income (minimum monthly salary (MMS): EUR 736 /USD 816 in 2018), middle income (2–4 times the MMS), and high income (>4 times the MMS). Tobacco history was divided into 4 categories: current smokers, occasional smokers (≤1 cigarette/day), ex-smokers, and never-smokers. Additionally, the daily cigarette use of current and ex-smokers was registered (<5 cigarettes/day, 5–10 cigarettes/day, 10–20 cigarettes/day, and >20 cigarettes/day). Standard beverage units (SBUs) were used to assess alcohol consumption (1 SBU = 10 g of ethanol), and moderate alcohol intake was defined as stated above. Participants were categorized as never having consumed alcohol, former drinkers, moderate drinkers, and heavy drinkers. Comorbidities were defined as a self-reported diagnosis of type 2 diabetes mellitus, high blood pressure, or cardiovascular disease by a physician or other health care provider. Participants were divided into 3 categories based on how often they engaged in moderate physical activity in the previous month: no physical activity, 1–2 times per week, or ≥3 times per week. Moderate physical activity was defined as brisk walking for 30 min or more each time.

### 2.8. Statistical Analysis

Data were collected through the Excel Corporation program (Microsoft, Redmond, WA, USA). Numerical variables were described according to their distribution as mean and standard deviation or median and minimum–maximum, whereas categorical variables were described by frequency and percentage. The chi-square test was used to assess bivariate associations between categorical variables. The relationships between numerical and categorical variables were evaluated based on the distribution of the numerical variables at each level of the categorical variable. The Mann–Whitney or Kruskal–Wallis tests were used for non-parametric distributions, and Student’s *t*-tests or ANOVA were used for normal distributions.

Multivariate logistic regression models were used to analyze the association between Mediterranean diet adherence, type of diet, and vitamin C and the risk of HNC. Models were adjusted to account for educational level, monthly salary, tobacco history, daily cigarette intake, alcohol use, and physical activity. Age and sex were not accounted for in the models because they were balanced by design. The odds ratio with 95% confidence intervals was used as the measure of association. Statistical significance was defined as a *p*-value of < 0.05. All analyses were performed with the software SPSS Statistics version 26 (IBM Corporation, Armonk, NY, USA). 

## 3. Results

### 3.1. Characteristics of Study Participants

The total sample size included 101 patients with HNC (cases) and 101 controls (*n* = 202). The mean age of participants was 65.8 ± 9.9 years, and 68.3% were male (Table 1). Comparing cases to controls, the cases had a lower educational level and lower monthly income. Regarding smoking behaviors, 71.3% of patients with HNC had a history of tobacco smoking, with 58.4% being former smokers, whereas almost 50% of the controls reported ever smoking. In addition, a significantly higher percentage of cases (69%) than of controls (24%) (*p* = 0.001) reported a smoking history of more than 20 cigarettes per day (Table 1). Cases were also more likely to have higher levels of alcohol intake than patients without HNC (23.8% vs. 4%, respectively) (*p* < 0.0001). Conversely, more controls than cases (73.2% vs. 52.3%, respectively) reported engaging in regular physical exercise at least twice per week. Cases had a higher prevalence of cardiovascular disease, while controls had a higher prevalence of type 2 diabetes mellitus (DM-2) (Table 1). The cancer history of patients diagnosed with HNC can be found elsewhere [16]. 

### 3.2. Association between Adherence to the Mediterranean Diet and the Risk of Head and Neck Cancer

**Figure 1:**
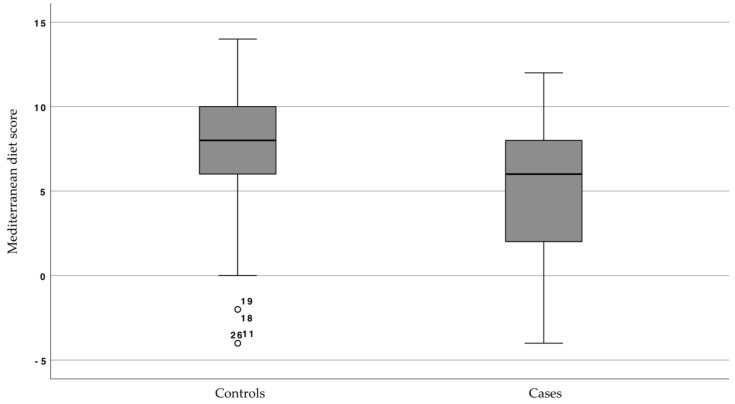
Distribution of the Mediterranean diet score in cases and controls.

The median diet score was significantly lower in cases than in controls (*p* < 0.001) (Table 2). Figure 1 shows that in 50% of cases the diet score varied between 2 and 8 (Q25–Q75), while in 50% of controls it varied between 6 and 10, with some outliers. Regarding the type of diet, the percentage of patients who maintained a healthy diet was significantly higher in controls (38.6%) than in patients with HNC (12.7%), who mainly followed a poor diet (46.8%) (*p* < 0.001) (Table 2).

Adherence to the Mediterranean diet was found to be significantly associated with a lower risk of HNC. After adjusting the multivariate logistic regression models, a higher adherence, indicated by a 1-unit increase in the diet score, was associated with a 12% reduction in the risk of HNC (OR = 0.88, 95% CI: 0.79–0.98) (Table 2). Additionally, having a healthy diet (10–14 points) was significantly associated with lower odds of HNC when compared with an unhealthy diet (OR = 0.29, 95% CI: 0.10–0.84) (Table 2).

### 3.3. Association between Types of Foods and Head and Neck Cancer Risk

As shown in Table 3, the intake of vegetables, fruits, whole grains, nuts, and eggs was higher in controls than in patients with HNC, although bivariate associations were not statistically significant. On the other hand, cases reported a significantly higher intake of milk and whole dairy products, salt, and industrial pastries compared with controls (*p* = 0.001) (Table 3). Multivariate logistic regression models showed that a moderate egg intake (<4 eggs/week) was the only type of food significantly associated with lower odds of head and neck cancer (OR = 0.26, 95% CI: 0.08–0.81) (Table 3). 

### 3.4. Association between Vitamin C Intake and the Risk of Head and Neck Cancer

Daily intake of vitamin C was significantly higher in the controls (81.3%) than in the cases (46.8%) (*p* < 0.001) (Table 4). Table 4 also shows that the primary source of vitamin C, in both the cases and controls, was natural sources, with a low percentage of patients taking vitamin supplements. Daily intake of vitamin C was significantly associated with a lower risk of head and neck cancer. After controlling for confounders, vitamin C was associated with an OR of 0.25 (95% CI: 0.10–0.62) when compared with those who did not take vitamin C on a daily basis (Table 4). 

## 4. Discussion

The results of this study showed that adherence to the Mediterranean diet, a healthy diet, and the daily intake of vitamin C are significant and inversely associated with the risk of head and neck cancer. In addition, moderate egg intake (<4 eggs/week) was the only type of food independently associated with a lower head and neck cancer risk. 

These findings are in line with previous published studies. It has been reported that adherence to the Mediterranean diet is associated with a 44% relative risk reduction of HNC compared with non-adherence (RR = 0.56, 95% CI: 0.44–0.72) [15]. Similarly, Giraldi et al. [18] reported a 36% decreased risk of HNC for each unit increase in the Mediterranean diet score (OR = 0.64, 95% CI: 0.58–0.71) and Saraiya et al. [19] a 13% increased risk of HNC for each unit decrease in the Mediterranean diet score (OR = 1.13, 95% CI: 1.02–1.25). The results from another study performed in Italy and Switzerland showed that an adherence between 66.7 and 100% was strongly associated with a lower risk of HNC (OR = 0.20, 95% CI: 0.14–0.28) [14]. In this study, a healthy diet, represented by a Mediterranean diet score of 10 or more points (10–14), was also strongly associated with a lower HNC risk (OR = 0.29, 95% IC: 0.10–0.84), which is equivalent to an adherence of 71.4% or more.

The results of this study are also in agreement with the recommendations of the World Cancer Research Fund and the American Institute for Cancer Research (WCRF/AICR) which recommend, for general cancer prevention, the maintenance of a healthy weight, physical activity, and a healthy diet rich in whole grains, fruits, and vegetables [3]. According to the World Health Organization (WHO), a healthy diet includes fruits, vegetables, legumes, nuts, and whole grains [20]. The Mediterranean diet is a dietary pattern mainly characterized by a high intake of vegetables, fruits, legumes, whole grains, nuts, and olive oil [13]. Its protective effects on cancer are attributed to the high contents of bioactive components with antioxidant and anti-inflammatory effects which counteract the inflammatory process and prevent oxidative damage to cellular DNA [13]. 

Maintaining a healthy lifestyle may prevent the onset of cancer [21]. A healthy lifestyle includes, among other things, being physically active, maintaining a healthy weight, eating a healthy diet, limiting alcohol consumption, and avoiding tobacco smoking. While there is strong evidence that tobacco, alcohol, and being overweight or obese increase the risk of HNC, there is limited but suggestive evidence that consuming non-starchy vegetables and coffee and choosing healthy dietary patterns may decrease the risk of HNC [3]. For other type of foods, micronutrients, and physical activity there is still no conclusions [3]. Particularly, head and neck cancers, depends heavily on tobacco smoking (population-attributable fraction (PAF) 65–80%), alcohol consumption (PAF 20–50%) and deficient intake of fruits and vegetables (45–56%), which may differ by site and cancer subtype [22]. Therefore, a healthy dietary pattern may be a strong and independent protective factor for head and neck cancers.

Regarding the types of foods, moderate egg intake (<4 eggs/week) was the only type of food significantly associated with a lower risk of HNC (OR = 0.26, 95% CI: 0.08–0.81). The impact of egg intake on general health is controversial, mainly because of its cholesterol content and potential role in cardio-metabolic events. Although high cholesterol levels are detrimental to health, dietary cholesterol has only a limited and clinically insignificant effect on blood cholesterol [23]. In fact, the effective contribution of dietary cholesterol on cardiovascular disease has been shown to be substantially nil and particularly attributed to saturated fatty acids and trans fats [24,25]. Increased dietary (exogenous) cholesterol intake has been shown to be associated with decreased endogenous cholesterol synthesis, possibly as a compensatory mechanism that maintains constant cholesterol homeostasis [26]. When considering dietary recommendations, recent guidelines have removed restrictions on total dietary cholesterol intake to 300 mg/day, and there is agreement on the recommendation of moderate egg intake as part of a healthy dietary pattern [25,27].

Eggs are a highly nutritious food; they are a rich source of protein, vitamins, and minerals, with a minimum of saturated fatty acids (1.56 mg/egg); particularly “rich” (when the recommended daily dose exceeds 30% of the nutrient level) in vitamin D, riboflavin, vitamin B12, biotin, and iodine; and a source of vitamin A, folate, choline, phosphorus, and selenium [25,28]. These micronutrients act as bioactive components with antioxidant, anti-inflammatory, immune-modulating, anticancer, and antihypertensive properties [29]. Egg yolk contains fat-soluble vitamins (A, D, E, and K), vitamin B12, carotenoid components, iron, and selenium, which are highly bioavailable and act as antioxidants promoting the neutralization of free radicals and, therefore, protecting against oxidative stress. Egg whites contain different proteins such as ovalbumin, ovotransferrin, ovomucin, and lysozyme that have immunoprotective, antimicrobial, and antioxidant action [29]. It has been shown that egg intake does not have a significant impact on inflammatory biomarkers (C-reactive protein, IL-6, TNF-α) [30]. Choline, present in egg yolk, has been associated with decreased systemic inflammatory markers in plasma levels, implicated in cardiovascular risk and carcinogenesis [27]. 

To date, only one systematic review has assessed the effect of egg intake on the risk of upper aero-digestive tract cancers. The findings showed that high egg intake (≥1 egg/day) was associated with an increased risk of oropharyngeal and laryngeal cancer (OR = 1.49, IC 95%: 1.35–1.64) compared with moderate egg consumption (0–20 g/day) [31]. Analyses by anatomical cancer sites showed that high egg intake was associated with an increased risk of oropharyngeal (OR = 1.88, 95% CI: 1.61–2.20) and laryngeal cancer (OR = 1.83, 95% CI: 1.45–2.32), whereas it was associated with a lower risk of oral cancer (OR = 0.78, 95% CI: 0.62–0.99). However, these findings were not confirmed by restricting the analysis to prospective cohort studies (OR = 0.86, IC 95%: 0.71–1.04) [31]; therefore, the findings must be interpreted with caution. The results from our study showed a beneficial effect of moderate egg intake on HNC risk. In 2018, the World Cancer Research Fund and the American Institute for Cancer Research (WCRF/AICR) concluded that there is limited evidence regarding egg intake and the risk of oral, pharyngeal, and laryngeal cancer; thus, it is not yet possible to determine whether its effect is beneficial, harmful, or has no impact on HNC risk [3].

Daily intake of vitamin C was strongly associated with a lower risk of HNC (OR = 0.25, IC 95%: 0.10–0.62). These findings are consistent with the available evidence. Previously, it has been shown that vitamin C intake has a protective effect on the risk of oral and pharyngeal cancer (OR = 0.54, 95% CI: 0.45–0.65) and laryngeal cancer (OR = 0.52, 95% CI: 0.40–0.68) [8]. The observed association has also been confirmed by a prospective cohort study, with a 20-year follow-up, where 144.8–153.3 mg/day of vitamin C was associated with an HNC relative risk reduction of 61% compared with the intake of 55.2–63.5 mg/day (RR = 0.39, 95% CI: 0.23–0.66) [32]. The protective effect is mainly related to its anti-inflammatory and antioxidant mechanisms, which counteract the inflammatory process and subsequent oxidative damage to DNA, which plays a significant role in the initiation and progression of cancer [8,32]. 

Regarding vitamin supplementation, the results from a pooled analysis showed a significant inverse association between vitamin C supplementation and HNC risk (OR = 0.76, 95% CI: 0.59–0.96), without a dose- or time-dependent relationship. The authors found no associations between the intake of multiple vitamin supplements or vitamin A, vitamin E, β-carotene, iron, selenium, or zinc supplements and the risk of HNC [33]. This pooled analysis included 7002 cases and 8383 controls, where the overall intake of vitamin supplementation was reported by 37.6% of cases and 48.3% of controls. Specifically, 7.7% of cases and 15% of controls reported vitamin C supplementation [33]. In our study, a similar proportion was observed in the cases (6.3%); however, in the controls, the proportion was considerably lower (4.4%). Recently, the Spanish Association of Nutrition and Dietetics published a study conducted in 2630 adults with the aim of assessing the prevalence of nutritional supplementation in the Spanish population. A total of 40% of the respondents reported vitamin supplementation and 31% specifically vitamin C supplementation [34]. Intake was significantly higher in women, younger people (26–35 years), those with higher education, and the physically active [34]. These demographic characteristics could explain the low percentage of vitamin C supplementation observed in our sample, which was mostly constituted of men (68.3%), those of advanced age (≥65 years) (56.4%), those with primary education (51.3%), and those who did not engage in regular physical activity (65.8%); therefore, our sample is not representative of the population that consumes vitamin C supplementation, which was expected.

There are several limitations of our findings. The study’s retrospective nature increases its susceptibility to selection bias, recall bias, and difficulties with accurately measuring exposure history. However, we designed a structured survey that allowed us to quantify the prognostic factors that affect the incidence of HNC and that were considered in the multivariate logistic regression models in order to reduce these sources of bias. The results must be interpreted with caution because current dietary habits were considered for both the cases and controls. Therefore, it is possible that the observed associations are underestimated or overestimated due to the modification of dietary habits after cancer diagnosis, which may improve in some cases or worsen in others depending on the morbidity associated with the cancer treatment. Despite this limitation, it was still possible to determine the beneficial effect of a healthy diet on the risk of HNC. The associations were strong for healthy diet, moderate egg, and daily vitamin C intake.

Even though the estimated ORs were adjusted for risk factors known to affect the incidence of HNC and the cases and controls were matched by age and sex, residual confounding cannot be ruled out. Despite being hospital-based, the controls came from the same geographic area and source as the cases. As they share the same selection procedures used to identify cases, these kinds of controls are frequently utilized in case–control studies because they result in a more efficient design.

## 5. Conclusions

Adherence to the Mediterranean diet and a healthy diet are associated with a lower risk of head and neck cancer. The type of diet and dietary patterns seem to have a greater impact on head and neck cancer risk than individual foods. Moderate egg intake (<4 eggs/week) is associated with a beneficial effect on head and neck cancer risk. Daily vitamin C intake is strongly associated with a lower risk of head and neck cancer. Overall, dietary patterns that emphasize a high intake of antioxidant and anti-inflammatory bioactive components may have a protective effect on the risk of head and neck cancer. 

## Figures and Tables

**Table 1:** Sociodemographic characteristics of cases and controls.

Characteristics	Total Sample (*n* = 202) *n* (%)	Cases (*n* = 101) *n* (%)	Controls (*n* = 101) *n* (%)	*p*-Value
**Sex**				
Male	138 (68.3)	69 (68.3)	69 (68.3)	
Female	64 (31.7)	32 (31.7)	32 (31.7)	
**Age (years) *(mean ± sd)***	65.8 ± 9.9	66 ± 10.2	65.5 ± 9.8	0.73
44–54 years	30 (14.9)	15 (14.9)	15 (14.9)	
55–64 years	58 (28.7)	29 (28.7)	29 (28.7)	
≥65 years	114 (56.4)	57 (56.4)	57 (56.4)	
**Education**				0.006
Without education	11 (5.6)	9 (9.4)	2 (2)	
Primary education	101 (51.3)	52 (54.2)	49 (48.5)	
Secondary education	43 (21.8)	23 (24)	20 (19.8)	
Higher education	42 (21.3)	12 (12.5)	30 (29.7)	
**Monthly income**				<0.0001
Low income	71 (39)	48 (59.3)	23 (22.8)	
Middle income	93 (51.1)	32 (39.5)	61 (60.4)	
High income	18 (9.9)	1 (1.2)	17 (16.8)	
**Smoking status**				0.002
Current smoker	28 (13.9)	12 (11.9)	16 (15.8)	
Occasional smoker	4 (2)	1 (1)	3 (3)	
Ex-smoker	91 (45)	59 (58.4)	32 (31.7)	
Never smoked	79 (39.1)	29 (28.7)	50 (49.5)	
**Cigarettes/day**				0.001
>20	47 (54)	40 (69)	7 (24.1)	
10–20	15 (17.2)	6 (10.3)	9 (31)	
5–10	11 (12.6)	6 (10.3)	5 (17.2)	
<5	14 (16.1)	6 (10.3)	8 (27.6)	
**Alcohol consumption**				<0.0001
Excessive alcohol intake	28 (13.9)	24 (23.8)	4 (4)	
Moderate alcohol intake	111 (55.2)	40 (39.6)	71 (71)	
Ex-consumer of alcoholic beverages	16 (8)	13 (12.9)	3 (3)	
Alcohol never consumed	46 (22.9)	24 (23.8)	22 (22)	
**Comorbidities**				
Diabetes mellitus 2	36 (17.8)	12 (11.9)	24 (23.8)	0.07
High blood pressure	88 (43.6)	39 (38.6)	49 (48.5)	0.16
Cardiovascular disease	32 (15.8)	21 (20.8)	11 (10.9)	0.05
**Physical activity**				0.01
≥3 times/week	64 (34.2)	26 (30.2)	38 (37.6)	
1–2 times/week	55 (29.4)	19 (22.1)	36 (35.6)	
No practice	68 (36.4)	41 (47.7)	27 (26.7)	

**Table 2:** Mediterranean diet score in cases and controls and adjusted logistic regression models (OR) for the association between adherence to the Mediterranean diet and head and neck cancer risk.

Variables	Cases (*n* = 101) *n* (%)	Controls (*n* = 101) *n* (%)	*p*-Value ^‡^	Crude OR (95% CI)	Adjusted OR ^†^ (95% CI)
Mediterranean diet score (0–14 points) *	6 (−4–12)	8 (−4–14)	<0.001	0.86 (0.79–0.93)	0.88 (0.79–0.98)
**Type of diet**					
Healthy diet (10–14 points)	10 (12.7)	39 (38.6)	<0.001	0.16 (0.07–0.36)	0.29 (0.10–0.84)
Regular diet (5–9 points)	32 (40.5)	40 (39.6)		0.48 (0.23–0.96)	0.77 (0.32–1.86)
Unhealthy diet (≤4 points)	37 (46.8)	22 (21.8)		Reference	Reference

* Median (minimum–maximum). ^‡^ Bivariate associations (chi-square test). ^†^ Adjusted for education, monthly income, smoking status, alcohol consumption, physical activity, and comorbidities.

**Table 3:** Types of food distribution in cases and controls, and logistic regression models (OR) for its association with head and neck cancer.

Variables	Cases (*n* = 101) *n* (%)	Controls (*n* = 101) *n* (%)	*p*-Value ^‡^	Crude OR (95% CI)	Adjusted OR ^†^ (95% CI)
Vegetables and greens (>5 days/week)	48 (67.6)	79 (78.2)	0.12	0.55 (0.27–1.12)	0.81 (0.31–2.10)
Fruits (>1 serving/day)	46 (64.8)	78 (77.2)	0.07	0.52 (0.26–1.03)	0.55 (0.21–1.45)
Whole-grain foods (≥1 serving/day)	33 (46.5)	49 (48.5)	0.79	0.96 (0.51–1.81)	1.25 (0.55–2.87)
Legumes (≥2 days/week)	60 (84.5)	77 (76.2)	0.18	1.55 (0.69–3.49)	2.98 (0.96–9.28)
Nuts (≥2 days/week)	26 (36.6)	51 (50.5)	0.07	0.72 (0.38–1.37)	0.82 (0.34–1.98)
Olive oil (daily)	69 (97.2)	98 (97)	0.95	1.12 (0.18–6.93)	2.14 (0.19–23.9)
Fish or shellfish (≥2 days/week)	55 (77.5)	73 (73.3)	0.44	1.30 (0.63–2.67)	1.65 (0.59–4.63)
Eggs (<4 eggs/week)	54 (76.1)	87 (86.1)	0.09	0.49 (0.22–1.13)	0.26 (0.08–0.81)
Red meat or sausages (<3 days/week)	49 (69)	70 (69.3)	0.97	0.99 (0.50–1.96)	1.32 (0.48–3.60)
Milk and whole dairy products (>2 days/week)	53 (74.6)	51 (50.5)	0.001	3.24 (1.65–6.38)	2.49 (0.94–6.61)
Add salt to meals at the table	33 (46.5)	23 (22.8)	0.001	3.39 (1.68–6.86)	1.35 (0.50–3.68)
Industrial pastries (>2 days/week)	21 (29.6)	8 (7.9)	<0.001	5.49 (2.06–14.7)	1.93 (0.53–7.09)
Industrial soft drinks (not light) (>2 days/week)	9 (12.7)	9 (8.9)	0.43	1.05 (0.37–2.96)	0.43 (0.07–2.66)

^‡^ Bivariate associations (chi-square test). ^†^ Adjusted for education, monthly income, smoking status, alcohol consumption, physical activity, comorbidities, and type of diet.

**Table 4:** Intake of vitamin C in cases and controls, and logistic regression models (OR) for its association with head and neck cancer.

Variables	Cases (*n* = 101) *n* (%)	Controls (*n* = 101) *n* (%)	*p*-Value ^‡^	Crude OR (95% CI)	Adjusted OR ^†^ (95% CI)
**Vitamin C**					
Daily intake of vitamin C *(Natural sources or vitamin supplements)*	37 (46.8)	74 (81.3)	<0.001	0.24 (0.12–0.49)	0.25 (0.10–0.62)
Natural sources	36 (45.6)	74 (81.3)	<0.001	0.24 (0.12–0.49)	0.25 (0.10–0.62)
Vitamin supplements	5 (6.3)	4 (4.4)	0.57	1.35 (0.33–5.64)	2.33 (0.44–12.3)

^‡^ Bivariate associations (chi-square test). ^†^ Adjusted for education, monthly income, smoking status, alcohol consumption, physical activity, comorbidities, and type of diet.

## Data Availability

Data are contained within the article.
